# Structural and Electronic Properties of Heterostructures Composed of Antimonene and Monolayer MoS_2_

**DOI:** 10.3390/nano10122358

**Published:** 2020-11-27

**Authors:** Congcong Zhou, Xiaodan Li, Taotao Hu

**Affiliations:** 1 College of Science, University of Shanghai for Science and Technology, Shanghai 200093, China; 172282026@st.usst.edu.cn; 2 School of Physics, Northeast Normal University, Changchun 130024, China; hutt262@nenu.edu.cn

**Keywords:** Sb/MoS_2_ hetero-bilayer, electronic properties, type-II band alignment

## Abstract

Antimonene is found to be a promising material for two-dimensional optoelectronic equipment due to its broad band gap and high carrier mobility. The van der Waals heterostructure, as a unique structural unit for the study of photoelectric properties, has attracted great attention. By using ab initio density functional theory with van der Waals corrections, we theoretically investigated the structural and electronic properties of the heterostructures composed of antimonene and monolayer MoS_2_. Our results revealed that the Sb/MoS_2_ hetero-bilayer is an indirect semiconductor with type-II band alignment, which implies the spatial separation of photogenerated electron–hole pairs. Due to the weak van der Waals interlayer interactions between the adjacent sheets of the hetero-bilayer systems, the band structures of isolated antimonene and monolayer MoS_2_ are preserved. In addition, a tunable band gap in Sb/MoS_2_ hetero-bilayer can be realized by applying in-plane biaxial compressing/stretching. When antimonene and monolayer MoS_2_ are stacked into superlattices, the indirect semiconductors turn into direct semiconductors with the decreased band gaps. Our results show that the antimonene-based hybrid structures are good candidate structures for photovoltaic devices.

## 1. Introduction

Due to its unique properties and potential applications, graphene, a two-dimensional (2D) material honeycomb lattice composed of C atoms, has caused a tremendous upsurge of scientific interest [1,2,3,4]. Graphene and its polymers have excellent photoelectric and chemical properties, which are widely used in transistor and photoelectric devices [5,6,7,8,9,10,11,12]. However, graphene is a zero-band gap semiconductor with poor current switching ability, which limits its application in photoelectric devices [13]. Since the band gap is an essential property for logic circuit of field-effect transistors [14], we should find the substitutional materials for graphene. For example, boronnitride (BN) is a wide-band gap insulating material with good electrical insulation, high thermal conductivity and excellent lubricant properties [15,16,17]. Molybdenum disulfide (MoS_2_), a typical layered-transition metal halide material, has been used widely in gas-sensing fields due to its high chemical response and excellent electronic properties [18,19,20]. Through mechanical exfoliation, the single layers of antimony can be readily produced [21,22]. The bilayer antimonene is shown to have enhanced stability and superconductivity, which is essential for the Li-ion battery [23,24,25]. However, with the rapid development of optoelectronic and new energy fields, a higher demand has been presented in 2D materials. Since single-structure materials are unable to meet the increasing demand of the material field, it is particularly important to study hybrid systems composed of different 2D materials.

Recent studies have shown that the two-dimensional components of hybrid systems could complement each other, which would provide more opportunities for achieving the desired optoelectronic properties. The electronic properties of hybrid systems change with the system’s ingredients, resulting from the combination of van der Waals interactions and interlayer characteristics. For example, due to the introduction of MoS_2_, the charge mobility and the energy band of MoS_2_/Si and MoS_2_/Ge hybrid systems are increased, enhancing the application of electronic and optical properties in multifunctional devices [26,27,28,29]. A strong interlayer coupling between MoS_2_ and graphene was found in MoS_2_/graphene heterostructures [30,31]. The coupling effect of BN with other components (graphene, graphite or metal) is strong, which means BN-based heterostructures can be used as a stable, high-purity and high-performance nano-scale dielectric material [32,33,34,35]. Moreover, a type-II band alignment was found in GaSe/MoSe_2_ [36], black phosphorene/MoS_2_ [37], Sb/graphene [38] and Sb/selenide [39] heterostructures. The type-II band arrangement can transfer holes and electrons to different materials, thus reducing the exciton recombination and increasing the exciton lifetime.

In contrast to flat graphene, antimonene is a pleated semiconductor with a finite band gap value and higher carrier mobility. In addition, the band gap of antimonene changes with the thickness, and it has higher chemical stability in the air. Considering the high sensitivity of antimonene, in this work, using density functional calculations with van der Waals correction, we systematically investigated the structural and electronic properties of hybrid systems composed of MoS_2_ and antimonene. We focus on the charge distribution and bonding nature in the hybrid systems, which would be helpful for a deeper understanding of common rules in electronic properties and type-II band gap control of hybrid systems. Our project helps in moving from theoretical results to experimental simulation, which can provide a theoretical foundation for the application of monolayer MoS_2_-based optoelectronic devices.

## 2. Methods

First-principles calculations were performed using the projector augmented wave (PAW) method within the density functional theory (DFT) [40] as implemented in the Vienna ab initio simulation package (VASP) [41,42]. The generalized gradient approximation (GGA) was used to deal with the electron interaction correlation potential [43]. In addition, in order to better explain the weak van der Waals interactions, which is crucial for layered heterostructures, all calculations in this work were performed using the PBE-D2 method [44,45]. In order to avoid the corresponding interlayer coupling, a vacuum layer of 15 Å in the *z* direction was set between two adjacent hetero-bilayers. The Brillouin zone integrations were approximated using the special K-point sampling of the Monkhorst–Pack scheme [46,47] with a Γ-centered 15 × 15 × 1 grid. Wave functions were expanded in plane waves up to a kinetic energy cutoff of 500 eV. The cell parameters and the atomic coordinates of all layered heterostructures are fully relaxed until the change of the energy and the force reaches 10^−5^ eV per supercell and 10^−2^ eV/Å, respectively. Although spin-polarized calculations were performed, the results indicated that all the layered heterostructures do not exhibit magnetism at their equilibrium states.

## 3. Results and Discussion

**Figure 1:**
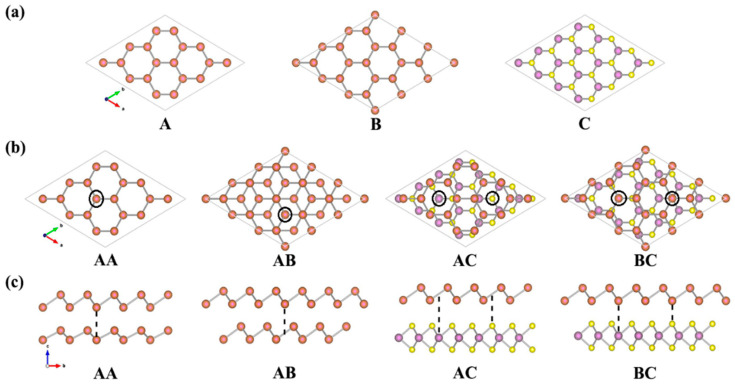
(Color online) (**a**) Top view of the antimonene and MoS_2_ monolayer; (**b**) top view and (**c**) side view of the bilayer antimonene and the Sb/MoS_2_ hetero-bilayer. Sb, Mo and S atoms are represented by red, purple and yellow balls, respectively.

According to our results performed using the PBE-D2 method, the lattice constants of a free-standing monolayer MoS_2_ and antimonene are 3.19 Å and 4.06 Å, respectively, which are in good agreement with the reported value [48,49,50,51,52]. Although the lattice constant of antimonene differs significantly from that of single-layer MoS_2_, they do share the same primitive cell of a hexagonal structure. In order to reduce the lattice mismatch between the antimonene and the MoS_2_ monolayer, the hybrid system was modeled with a supercell consisting of 3 × 3 unit cells of antimonene and 4 × 4 unit cells of MoS_2_ monolayer in the x-y plane. The lattice constants of 3 × 3 antimonene and 4 × 4 MoS_2_ are 12.18 Å and 12.76 Å, respectively, which leads to a 4.6% lattice mismatch initially in the Sb/MoS_2_ hetero-bilayer. Compared with the heterostructures investigated previously, this lattice mismatch value is relatively small [53,54,55]. The optimized structure of the antimonene and single layer MoS_2_ are shown in Figure 1. Both A and B represent the geometric structure of antimonene, and B was obtained by rotating the structure A by 60°. C is the geometric structure of MoS_2_ monolayer. Through the weak VDW force, the antimonene can form a multilayer structure. The bilayer antimonene structures labeled as AA and AB are shown in Figure 1b. AA is a hexagonal stacking model, in which all Sb atoms in the upper layer (A) are located above the Sb atom in the lower layer (A). AB is the Bernal stacking model, in which the B layer is constructed from a rotation of the A layer by 60°. For the hetero-bilayer structure composed of antimonene and the MoS_2_ monolayer (marked as Sb/MoS_2_), two types of stacking models were considered: (1) hollow-site stacking (denoted by AC), the center of antimony hexagons on top of one of the Mo or S atoms of MoS_2_ monolayer; and (2) top-site stacking (denoted by BC), a Sb atom of antimonene on top of one of the Mo or S atoms of the MoS_2_ monolayer. The side views of the bilayer antimonene and the Sb/MoS_2_ hetero-bilayer are shown in Figure 1c.

The optimized structural parameters of the bilayer antimonene are shown in Table 1. The lattice constant “*a*” of the AA and AB stacking is 12.73 Å and 12.28 Å, respectively. Compared to the isolated antimonene, the AA stacking structure is stretched by 4.5% and the AB stacking structure is stretched by 0.8%. The results show that there is a significant difference in the lattice constant between two stacking bilayer antimonene, which is caused by the increased coupling between layers. A shorter interlayer distance was found in the AB stacking bilayer antimonene, indicating that the interlayer interaction of the AB stacking structure is stronger. This can also explain why the amplitude of buckling (∆) of the AA stack is slightly larger than the AB stack. To discuss the relative stabilities of the bilayer systems, all the binding energy ($E_{b}$) in this paper is defined as:

$$E_{b}=\left(E_{A}+E_{B}\right)-E_{AB}$$

where $E_{A}$ and $E_{B}$ represent the total energies of the isolated monolayers and $E_{AB}$ is the total energy of the heterostructure. The binding energies are given per unit cell. As shown in Table 1, the binding energy of bilayer antimonene and Sb/MoS_2_ hetero-bilayers are positive. This indicates that the layer stack is an exothermic process; in other words, the two-layer system discussed in this paper can exist stably. In addition, the small binding energy also indicates the weak interaction between the antimonene and MoS_2_ layers. The binding energy of bilayer antimonene optimized via the PBE-D2 method is 2.275 eV and 3.513 eV for the AA and AB stacking model, respectively (see Table 1), indicating that the interlayer interaction of the AB stacking is stronger than that of the AA stacking.

In order to better understand the properties of antimonene-based hybrid structures, we also studied the hetero-bilayer (labeled as Sb/MoS_2_) and superlattice (labeled as Sb/MoS_2_) composed of antimonene and monolayer MoS_2_. For hybrid systems, to quantitatively characterize the interlayer interaction, the binding energy between the antimonene and MoS_2_ sheet was calculated using the adsorption energy formula. $E_{AB}$ is the total energy of the Sb/MoS_2_ hetero-bilayer and $E_{A}$/$E_{B}$ is the total energy of the isolated Sb sheet or monolayer MoS_2_. Similar to bilayer antimonene, for hybrid systems, we also considered two representative arrangements of antimonene on monolayer MoS_2_ (AC and BC). The optimized lattice constant “*a*” of the Sb/MoS_2_ hetero-bilayer is 12.67 Å and 12.66 Å, respectively. Judging from the optimized structural parameters and electronic structures of hetero-bilayer Sb/MoS_2_, there is no big difference between the AC and BC stacking structures (shown in Figure 1). These results indicate that the energetics of antimonene on MoS_2_ are not sensitive to the stacking models, which is similar to that of previous MoS_2_-based hybrid systems [29,30,31]. Compared to the corresponding isolated sheet, the Sb layer in the Sb/MoS2 hetero-bilayer is stretched by 3.94% (from 4.06 to 4.22 Å), while the MoS_2_ layer in the Sb/MoS_2_ heterogeneous double is compressed by 0.63% (from 3.19 to 3.17 Å).

**Figure 2:**
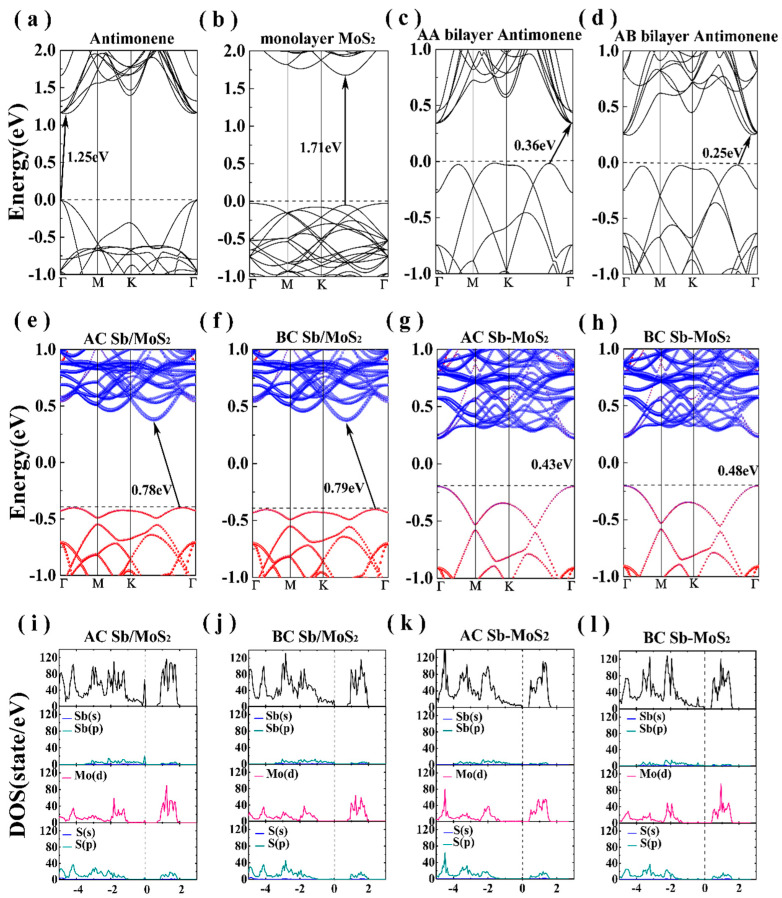
Band structures of (**a**) antimonene, (**b**) MoS_2_ monolayer, (**c**) AA bilayer antimonene, (**d**) AB bilayer antimonene, (**e**) AC Sb/MoS_2_ hetero-bilayer, (**f**) BC Sb/MoS_2_ hetero-bilayer, (**g**) AC Sb/MoS_2_ superlattice and (**h**) BC Sb/MoS_2_ superlattice. The density of states of (**i**) AC Sb/MoS_2_ hetero-bilayer, (**j**) BC Sb/MoS_2_ hetero-bilayer, (**k**) AC Sb/MoS_2_ superlattice and (**l**) BC Sb/MoS_2_ superlattice. The black dotted line indicates the Fermi level.

Figure 2 illustrates the electronic band structures of the bilayer systems. For monolayer antimonene, the maximum valence band (VBM) is located at the high symmetry Γ point, and the conduction band minimum (CBM) is located between the high symmetry point Γ and M. This indicates that the Sb monolayer is an indirect band gap semiconductor with a band gap of 1.25 eV, which is in good agreement with the reported value [21,22,23,24,25]. Moreover, for the MoS_2_ monolayer (shown in Figure 2b), both VBM and CBM are located between the high symmetry points Γ and M, suggesting that the MoS_2_ monolayer is a direct band gap semiconductor. The electronic band structures of bilayer antimonene with two different stacking models are shown in Figure 2c,d. We can see that the VBM of the AA stacked is located between the high symmetry points Γ and K, and the CBM is located at the high symmetry point Γ from Figure 2c. However, the CBM and VBM of the AB stacked are both located between the high symmetry points Γ and M. Therefore, the bilayer antimonene is an indirect band gap semiconductor with a band gap of 0.36 eV (AA stacking) and 0.25 eV (AB stacking). The band gap value of the bilayer antimonene is reduced compared to the Sb monolayer, which is consistent with other van der Waals layered structures [22,23,24,25]. Compared to the Sb monolayer, the band gap value of the bilayer antimonene is significantly sunken regardless of the model stack. This facilitates the charge transfer between layers and realizes the application of Sb in photovoltaic cells.

The projected band structures of hetero-bilayer Sb/MoS_2_ are shown in Figure 2e,f, the red and blue circles representing the contributions of Sb and MoS_2_ to the eigenstate, respectively. We can clearly see that the band structure of the Sb/MoS_2_ heterostructure is a simple superposition of the components. There is a simple coupling effect between Sb and MoS_2_ layers so that the electronic structure of Sb and MoS_2_ monolayer can be preserved. Both for AC and for BC stacking, the CBM and VBM are located between the high symmetry points Γ and M, indicating that the Sb/MoS_2_ hetero-bilayer is an indirect band gap semiconductor. In addition, the VBM of the Sb/MoS_2_ heterostructure is mainly distributed from Sb, while the CBM is localized in MoS_2_. Therefore, the Sb/MoS_2_ is a type-II hetero-bilayer structure. In the Sb/MoS_2_ type-II energy band alignment, MoS_2_ acts as an electron acceptor and Sb acts as an electron donor. According to the density of states (DOS) of the Sb/MoS_2_ hetero-bilayer shown in Figure 2i,j, the energy band near the Fermi level originates from the electron hybridization of the *p* orbital of Sb and the *d* orbital of Mo. Under the action of the VBM shift, the electrons in Sb can be easily transferred to the conduction band of the MoS_2_ layer. At the same time, the holes of the MoS_2_ layer can be easily transferred to the valence band of the Sb layer. Due to the electron and hole transfer, the electron–hole pairs of the Sb/MoS_2_ heterostructure decrease. This will effectively extend the life of the optoelectronics, thereby improving the performance of electronic and optoelectronic devices.

The band structures of Sb/MoS_2_ superlattice are shown in Figure 2g,h. The superlattices with two different stackings are all shown to be direct band gap semiconductors. Compared with the hetero-bilayer Sb/MoS_2_, the CBM of the Sb/MoS_2_ superlattice is still all contributed by MoS_2_, while the VBM is occupied by Sb and MoS_2_. The total and partial DOS of Sb/MoS_2_ superlattice are shown in Figure 2k,l. Within each monolayer of MoS_2_, there is strong hybridization between the Mo-*d* orbitals and the S-*s* orbitals, and the Mo-S bond could preserve its covalent bonding. Compared with the Sb/MoS_2_ hetero-bilayer (Figure 2i,j), we can clearly see that the contribution of antimonene in the valence band decreases, and the conduction band moves close to the Fermi level, leading to a decreased band gap in the Sb/MoS_2_ superlattice. By controlling the number of layers in heterostructures, antimonene-based hybrid structures could realize a transformation from a type-I semiconductor to a type-II semiconductor. The results of this work provide a promising way to design applications in future antimonene-based nanodevices with controlled band gaps.

**Figure 3:**
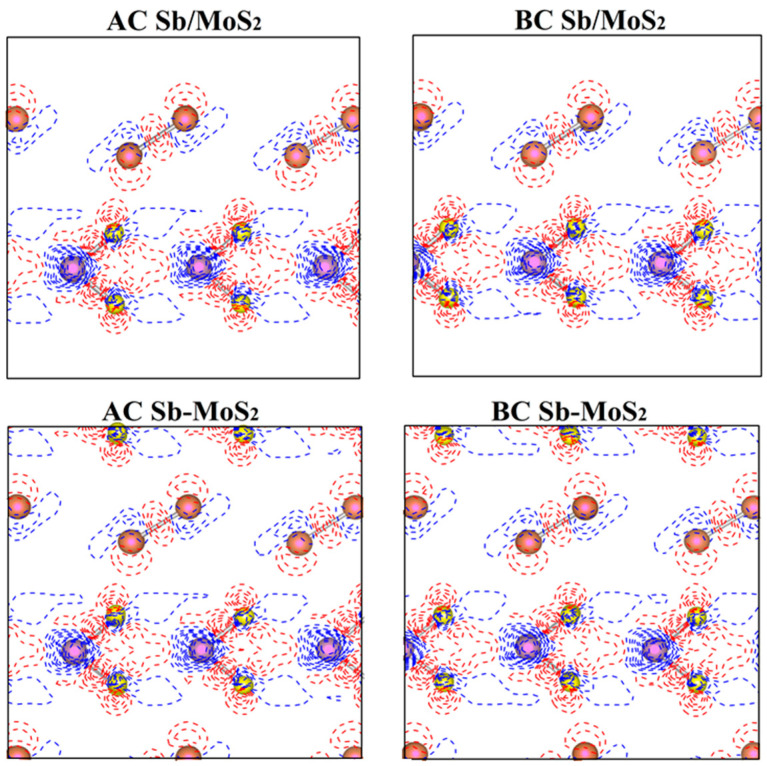
Deformation charge density ($\Delta \rho (\vec{r})$ for hybrid systems of planes perpendicular to the atomic layers and passing through Mo-S and Sb-Sb bonds. The red solid line and the blue dashed line indicate $\Delta \rho (\vec{r})$ < 0 and $\Delta \rho (\vec{r})$ < 0, respectively.

In order to better study the bonding nature and charge transfer of hybrid systems, we calculated the difference in charge density of planes perpendicular to the atomic layers and passing through Mo-S and Sb-Sb bonds (shown in Figure 3). The differential charge density ($\Delta \rho (\vec{r})$) here is defined as:

$$\Delta \rho (\vec{r})=\rho (\vec{r})-\sum_{\mu }\rho_{atom}(\vec{r}-{\vec{R}}_{\mu })$$

where $\rho (\vec{r})$ represents the total charge density of the heterostructure system and $\sum_{\mu }\rho_{atom}(\vec{r}-{\vec{R}}_{\mu })$ is the superposition of atomic charge densities. The red solid line ($\Delta \rho (\vec{r})$ > 0) and the blue dashed line ($\Delta \rho (\vec{r})$ < 0) indicate the increase and decrease in the electron density after bonding, respectively. All the charge densities here are calculated using the same supercell for the heterostructures. As shown in Figure 3, within each atomic layer, the atomic bonding of Mo-S and Sb–Sb could preserve their covalent bonding, indicating that the interactions within each 2D sheet in heterostructures are not affected significantly by the introduction of foreign 2D materials. However, the weak interaction between layers will lead to a simple coupling effect between Sb and MoS_2_ layers. To clarify the specific reasons, the Bader analysis for the heterostructures was performed to calculate the charge transfer between the antimonene and MoS_2_ monolayer. According to the data in Table 2, we found that electrons are transferred from the Sb layer to the neighboring MoS_2_ layer (at the junction of MoS_2_ and Sb layers). However, the amount of charge transfer is very small (less than one electron). This charge is newly distributed after the formation of the Sb/MoS_2_ heterostructures, which hinders the recombination of electron–hole pairs and balances the system.

**Figure 4:**
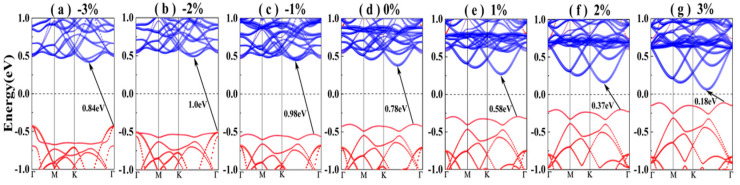
The electronic band structures of the Sb/MoS_2_ hetero-bilayer under different in-plane biaxial strain: (**a**) *ε* = −3%, (**b**) *ε* = −2%, (**c**) *ε* = −1%, (**d**) *ε* = 0%, (**e**) *ε* = 1%, (**f**) *ε* = 2%, (**g**) *ε* = 3%. Contribution from antimonene and MoS_2_ monolayer to the band structures of the bilayers are shown with red and blue circle dots, respectively. The Fermi level is set to 0 eV.

Compared to the isolated antimonene and MoS_2_ monolayer, the Sb and MoS_2_ layers in the hetero-bilayer have to be compressed or stretched to match each other. Thus, in order to get further insight into the effect of the in-plane biaxial strain on the hetero-bilayer systems, we systematically studied the electronic band structures and differential charge density of compressed (from −1% to −8%) and stretched (from 1% to 4%) Sb/MoS_2_ hetero-bilayers. The in-plane biaxial strain (*ε*) is defined as:

$$\varepsilon =\frac{a-a_{0}}{a}\times 100\%$$

where *a* and *a*_0_ are the strained and unstrained lattice constants, respectively. The electronic band structures of the Sb/ MoS_2_ hetero-bilayer under different strains are shown in Figure 4, in which the band contribution from the antimonene and MoS_2_ monolayer are shown with blue and red dots, respectively. The band structure of the unstrained Sb/MoS_2_ hetero-bilayer is also depicted in the middle of Figure 4 for comparison. For Sb/MoS_2_ hetero-bilayers, the compressed (less than −8%) or stretched (less than 4%) systems are still semiconductors with indirect band gaps. However, it can be seen that the value of band gaps can be varied by compressing or stretching the lattice constants (shown in Table 3). A maximum band gap of 1.00 eV appears at the Sb/MoS_2_ hetero-bilayer with a compressed strain of −2%. For stretched Sb/MoS_2_ hetero-bilayers, the magnitude of band gaps decreases monotonically with an increase in lattice parameters. The size of the strain-induced band gap of Sb/MoS_2_ hetero-bilayers is in the range of 0.10~1.00 eV. As the lattice constant is decreased, the band moves toward the deeper energy level. When a compressive strain of −8% is applied, the Sb/MoS_2_ hetero-bilayer is metallic. Hence, through compressing or stretching, the hetero-bilayers realize the transformation from a metal to an indirect band gap semiconductor.

## 4. Conclusions

In summary, the electronic structures and properties of hybrid systems composed of antimonene and monolayer MoS_2_ were studied through density functional theory calculations. The Sb/MoS_2_ hetero-bilayer and Sb/MoS_2_ superlattice are both type-II band alignment, which implies the spatial separation of photogenerated electron–hole pairs. The Sb/MoS_2_ hetero-bilayers are indirect band gap semiconductors, while the Sb/MoS_2_ superlattices were shown to be direct band gap semiconductors with decreased band gaps. Due to the weak van der Waals interlayer interactions between layers, the electronic properties of each component can be preserved in the hybrid systems. In addition, the band gap of the Sb/MoS_2_ hetero-bilayer can be effectively controlled by the biaxial strain, which improves its electronic performance in nanodevices. Our results show that antimonene-based hybrid systems are promising candidates for photovoltaic devices.

## Figures and Tables

**Table 1:** Geometries and binding energies of antimonene and MoS_2_ monolayer, antimonene bilayer, Sb/MoS_2_ hetero-bilayers and Sb/MoS_2_ superlattice, including the bond length, interlayer distance of stacked layer structure (h) and the amplitude of bucking of the antimonene layers (Δ*_Sb_*).

System		a (Å)	E_b_ (eV)	d_Sb–Sb_ (Å)	d_Mo-S_ (Å)	h (Å)	Δ_Sb_ (Å)
Antimonene		12.18	/	2.88	/	/	0.110
Monolayer MoS_2_		12.76	/	/	/	/	/
bilayer antimonene	AA	12.73	2.275	2.95	/	3.03	0.110
	AB	12.28	3.513	2.88	/	2.90	0.107
Sb/MoS_2_ hetero-bilayer	AC	12.67	1.377	2.92	2.42	3.41	0.074
	BC	12.66	1.378	2.92	2.41	3.41	0.075
Sb/MoS_2_ superlattice	AC	12.67	3.612	2.92	2.42	3.29	0.137
	BC	12.66	3.661	2.91	2.40	3.34	0.147

**Table 2:** Bader analysis for the heterostructures.

System		Gain/Loss Electrons	
		Sb Layer	MoS_2_ Layer
Sb/MoS_2_ hetero-bilayer	AC	−0.045	0.026
	BC	−0.045	0.025
Sb/MoS_2_ superlattice	AC	−0.099	0.056
	BC	−0.098	0.055

**Table 3:** Band gaps of compressed/stretched Sb/MoS_2_ hetero-bilayer.

strains	−8%	−7%	−6%	−5%	−4%	−3%	−2%	−1%	0%	1%	2%	3%	4%
band gap (eV)	metal	0.10	0.31	0.51	0.69	0.84	1.00	0.98	0.78	0.58	0.37	0.18	metal

## References

[1] Xie C., Mak C.H., Tao X.M., Yan F. (2017). Photodetectors Based on Dwo-Dimensional Layered Materials Beyond Graphene. Adv. Funct. Mater..

[2] Wu J.B., Lin M.L., Cong X., Liu H.N., Tan P.H. (2018). Raman Spectroscopy of Graphene-Based Materials and Its Applications in Related Devices. Chem. Soc. Rev..

[3] Yu X.W., Cheng H.H., Zhang M., Zhao Y., Qu L.T., Shi G.Q. (2017). Graphene-Based Smart Materials. Nat. Rev. Mater..

[4] Lu X.B., Stepanov P., Yang W., Xie M., Aamir M.A., Das I., Urgell C., Watanabe K., Taniguchi T., Zhang G. (2019). Superconductors, Orbital Magnets and Correlated States in Magic-Angle Bilayer Graphene. Nature.

[5] Abraham J., Vasu K.S., Williams C.D., Gopinadhan K., Su Y., Cherian C., Dix J., Prestat E., Haigh S.J., Grigorieva I.V. (2017). Tuneable Sieving of Ions Using Graphene Oxide Membranes. Nat. Nanotechnol..

[6] Mo R.W., Rooney D., Sun K., Yang H.Y. (2017). 3D Nitrogen-Doped Graphene Foam with Encapsulated Germanium/Nitrogen-Doped Graphene Yolk-Shell Nanoarchitecture for High-Performance Flexible Li-Ion Battery. Nat. Commun..

[7] Li X.L., Zhi L.J. (2018). Graphene Hybridization for Energy Storage Applications. Chem. Soc. Rev..

[8] Lee I.H., Yoo D., Avouris P., Low T., Oh S.H. (2019). Graphene Acoustic Plasmon Resonator for Ultrasensitive Infrared Spectroscopy. Nat. Nanotech..

[9] Geng P.B., Zheng S.S., Tang H., Zhu R.M., Zheng L., Cao S., Xue H.G., Pang H. (2018). Transition Metal Sulfides Based on Graphene for Electrochemical Energy Storage. Adv. Energy Mater..

[10] Xu Y., Zhang C.L., Zhou M., Fu Q., Zhao C.X., Wu M.H., Lei Y. (2018). Highly Nitrogen Doped Carbon Nanofibers with Superior Rate Capability and Cyclability for Potassium Ion Batteries. Nat. Commun..

[11] Ma J., Liu C.T. (2020). Using Negative Thermal Expansion Effect to Grow Spiny NiS/C Architecture and Its Application in Sodium-Ion Batteries. J. Alloys Compd..

[12] Cai Z.X., Su L., Wang H.J., Niu M., Gao H.F., Lu D., Li M.Z. (2020). Hydrophobic SiC@C Nanowire Foam with Broad-Band and Mechanically Controlled Electromagnetic Wave Absorption. ACS Appl. Mater. Interfaces.

[13] Wong K.L., Chuan M.W., Hamzah A., Rusli S., Alias N., Sultan M.S., Lim C.S., Tan M.L.T. (2020). Electronic Properties of Graphene Nanoribbons with Line-Edge Roughness Doped with Nitrogen and Boron. Physica E.

[14] Tang S.B., Wu W.H., Xie X.J., Li X.K., Gu J.J. (2017). Band Gap Opening of Bilayer Graphene by Graphene Oxide Support Doping. RSC Adv..

[15] Yang J., Tang L.S., Bao R.Y., Bai L., Liu Z.Y., Xie B.H., Yang M.B., Yang W. (2018). Hybrid Network Structure of Boron Bitride and Graphene Oxide in Shape-Stabilized Composite Phase Change Materials with Enhanced Thermal Conductivity and Light-to-Electric Energy Conversion Capability. Sol. Energy Mater. Sol. Cells..

[16] Ji M.X., Xia J.X., Di J., Liu Y.L., Chen R., Chen Z.G., Yin S., Li H.M. (2018). Graphene-Like Boron Nitride Induced Accelerated Charge Transfer for Boosting the Photocatalytic Behavior of Bi_4_O_5_I_2_ Towards Bisphenol a Removal. Chem. Eng. J..

[17] Li Q., Huo C.R., Yi K., Zhou L.L., Su L., Hou X.M. (2018). Preparation of Flake Hexagonal BN and Its Application in Electrochemical Detection of Ascorbic Acid, Dopamine and Uric Acid. Sens. Actuators B.

[18] Zhao J., Li N., Yu H., Wei Z., Liao M.Z., Chen P., Wang S.P., Shi D.X., Sun Q.J., Zhang G.Y. (2017). Highly Sensitive MoS_2_ Humidity Sensors Array for Noncontact Sensation. Adv. Mater..

[19] Liu W.J., Zhu Y.N., Liu M.L., Wen B., Fang S.B., Teng H., Lei M., Liu L.M., Wei Z.Y. (2018). Optical Properties and Applications for MoS_2_-Sb_2_Te_3_-MoS_2_ Heterostructure Materials. Photonics Res..

[20] Sangeetha M., Madhan D. (2020). Ultra Sensitive Molybdenum Disulfide (MoS_2_)/graphene Based Hybrid Sensor for The Detection of NO_2_ and Formaldehyde Gases by Fiber Optic Clad Modified Method. Opt. Laser Technol..

[21] Ares P., Aguilar-Galindo D., Rodriguez-San-Miguel D., Aldave D.A., Diaz-Tendero S., Alcami M., Martin F., Gomez-Herrero J., Zamora F. (2016). Mechanical Isolation of Highly Stable Antimonene Under Ambient Conditions. Adv. Mater..

[22] Pumera M., Sofer Z. (2017). 2D Monoelemental Arsenene, Antimonene, and Bismuthene: Beyond Black Phosphorus. Adv. Mater..

[23] Xie M.Q., Zhang S.L., Cai B., Gu Y., Liu X.H., Kan E.J., Zeng H.B. (2017). Van der Waals Bilayer Antimonene: A Promising Thermophotovoltaic Cell Material With 31% Energy Conversion Efficiency. Nano Energy.

[24] Wang X.X., Tang C.M., Zhou X.F., Zhu W.H., Fu L. (2019). The Good Performance of Bilayer β-Antimoneneas an Anode Material for the Li-ion Battery Study. Appl. Surf. Sci..

[25] Zhang J.J., Zhang Y., Dong S. (2018). Protective Layer Enhanced Stability and Superconductivity of Tailored Antimonene Bilayer. Phys. Rev. Mater..

[26] Alarawi A., Ramalingam V., Fu H.C., Purushothaman V., Yang R., He J.H. (2019). Enhanced Photoelectrochemical Hydrogen Production Efficiency of MoS_2_-Si Heterojunction. Opt. Express.

[27] Qiao S., Cong R.D., Liu J.H., Liang B.L., Fu G.S., Yu W., Ren K.L., Wang S.F., Pan C.F. (2018). A Vertically Layered MoS_2_/Si Heterojunction for an Ultrahigh and Ultrafast Photoresponse Photodetector. J. Mater. Chem. C.

[28] Son S.B., Kim Y., Cho B., Choi C.J., Hong W.K. (2018). Temperature-Dependent Electronic Charge Transport Characteristics at MoS_2_/p-type Ge heterojunctions. J. Alloys Compd..

[29] Li X.D., Yu S., Wu S.Q., Zhou S., Zhu Z.Z. (2014). Structural and Electronic Properties of Germanene/MoS_2_ Monolayer and Silicene/MoS_2_ Monolayer Superlattices. Nanoscale Res. Lett..

[30] Wan W., Li X.D., Li X.T., Xu B.B., Zhan L.J., Zhao Z.J., Zhang P.C., Wu S.Q., Zhu Z.Z., Huang H. (2016). Interlayer Coupling of a Direct Van der Waals Epitaxial MoS_2_/graphene Heterostructure. RSC Adv..

[31] Li X.D., Yu S., Wu S.Q., Wen Y.H., Zhou S., Zhu Z.Z. (2013). Structural and Electronic Properties of Superlattice Composed of Graphene and Monolayer MoS_2_. J. Phys. Chem. C.

[32] Han J.K., Du G.L., Gao W.W., Bai H. (2019). An Anisotropically High Thermal Conductive Boron Nitride/Epoxy Composite Based on Nacre-Mimetic 3D Network. Adv. Funct. Mater..

[33] Chittari B.L., Chen G.R., Zhang Y.B., Wang F., Jung J. (2019). Gate-Tunable Topological Flat Bands in Trilayer Graphene-Boron Nitride Moire Superlattices. Phys. Rev. Lett..

[34] Jiang Y.Y., Lin X., Low T., Zhang B.L., Chen H.S. (2018). Group-Velocity-Controlled and Gate-Tunable Directional Excitation of Polaritons in Graphene-boron Nitride Heterostructures. Laser Photonics Rev..

[35] Laturia A., Van de Put M.L., Vandenberghe W.G. (2018). Dielectric Properties of Hexagonal Boron Nitride and Transition Metal Dichalcogenides: From Monolayer to Bulk. NPJ 2D Mater. Appl..

[36] Pham K.D., Hieu N.N., Bui L.M., Phuc H.V., Hoi B.D., Tu L.T.N., Bach L.G., Ilyasov V.V., Amin B., Idress M. (2019). Vertical Strain and Electric Field Tunable Electronic Properties of Type-II Band Alignment C_2_N/InSe Van der Waals Heterostructure. Chem. Phys. Leet..

[37] You B.Q., Wang X.C., Zheng Z.D., Mi W.B. (2016). Black Phosphorene/Monolayer Transition-Metal Dichalcogenides as two Dimensional Van der Waals Heterostructures: A First-Principles Study. Phys. Chem. Chem. Phys..

[38] Zhang F., Li W., Dai X.Q. (2016). Effects of Interlayer Coupling on the Electronic Structures of Antimonene/Graphene Wan der Waals Heterostructures. Superlattices Microstruct..

[39] Shen N.F., Yang X.D., Wang X.X., Wang G.H., Wan J.G. (2019). Two-Dimensional Van der Waals Heterostructure of Indium Selenide/Antimonene: Efficient Carrier Separation. Chem. Phys. Lett..

[40] Sham L.J., Schlüter M. (1983). Density-Functional Theory of the Energy Gap. Phys. Rev. Lett..

[41] Kresse G., Furthmüller J. (1996). Efficient Iterative Schemes for Ab Initio Total-Energy Calculations Using a Plane-Wave Basis Set. Phys. Rev. B.

[42] Kresse G., Furthmüller J. (1996). Efficiency of Ab-Initio Total Energy Calculations for Metals and Semiconductors Using a Plane-Wave Basis Set. Comput. Mater. Sci..

[43] Perdew P.J., Burke K., Ernzerhof M. (1996). Generalized Gradient Approximation Made Simple. Phys. Rev. Lett..

[44] Perdew P.J., Zunger A. (1981). Self-Interaction Correction to Density-Functional Approximations for Many-Electron Systems. Phys. Rev. B.

[45] Grimme S. (2006). Semiempirical GGA-Dype Density Functional Constructed with A Long-Range Dispersion Correction. J. Comput. Chem..

[46] Chadi D.J. (1977). Special Points for Brillouin-Zone Integrations. Phys. Rev. B.

[47] Ramírez R., Böhm M.C. (1986). Simple Geometric Generation of Special Points in Brillouin-Zone Integrations. Two-Dimensional Bravais Lattices. Int. J. Quantum Chem..

[48] Chen B., Meng Y.H., Sha J.W., Zhong C., Hu W.B., Zhao N.Q. (2017). Preparation of MoS_2_/TiO_2_ Based Nanocomposites for Photocatalysis and Rechargeable Batteries: Progress, Challenges, and Perspective. Nanoscale.

[49] Wang S., Ren C.D., Tian H.Y., Yu J., Sun M.L. (2018). MoS_2_/ZnO Van der Waals Heterostructure as A High-Efficiency Water Splitting Photocatalyst: A First-Principles Study. Phys. Chem. Chem. Phys..

[50] Wu X., Shao Y., Liu H., Feng Z.L., Wang Y.L., Sun J.T., Liu C., Wang J.Q., Liu Z.L., Zhu S.Y. (2017). Epitaxial Growth and Air-Stability of Monolayer Antimonene on PdTe_2_. Adv. Mater..

[51] Shao Y., LIiu Z.L., Cheng C., Wu X., Liu H., Liu C., Wang J.O., Zhu S.Y., Wang Y.Q., Shi D.X. (2018). Epitaxial Growth of Flat Antimonene Monolayer: A New Honeycomb Analogue of Graphene. Nano Lett..

[52] Ares P., Palacios J.J., Abellan G., Gomez-Herrero J., Zamora F. (2017). Recent Progress on Antimonene: A New Bidimensional Material. Adv. Mater..

[53] Chen X.P., Meng R.S., Jiang J.K., Liang Q.H., Yang Q., Tan C.J., Sun X., Zhang S.L., Ren T.L. (2016). Electronic Structure and Optical Properties of Graphene/Stanene Heterobilayer. Phys. Chem. Chem. Phys..

[54] Águila E.J.C., Palacios P., Conesa C.J., Arriaga J., Wahnón P. (2016). Electronic Band Alignment at CuGaS_2_ Chalcopyrite Interfaces. Comput. Mater. Sci..

[55] Si F.J., Hu W., Tang F.L., Cheng Y.W., Xue H.T. (2017). Electronic and Optical Properties of the Wurtzite-ZnO/CH_3_NH_3_PbI_3_ Interface: First-Principles Calculations. J. Mater. Sci..

